# A Study of Cross-Protection between *Eimeria maxima* Immunovariants

**DOI:** 10.3390/pathogens13010066

**Published:** 2024-01-09

**Authors:** Mark C. Jenkins, Celia N. O’Brien, Carolyn C. Parker, Matthew S. Tucker

**Affiliations:** Animal Parasitic Diseases Laboratory, Beltsville Agricultural Research Center, Agricultural Research Service, USDA, Beltsville, MD 20705, USA; cnobrien26@yahoo.com (C.N.O.); carolyn.parker@usda.gov (C.C.P.); matthew.tucker2@usda.gov (M.S.T.)

**Keywords:** *Eimeria maxima*, immunovariability, protection, oocyst output

## Abstract

For reasons unknown, *Eimeria maxima* is unique among *Eimeria* species infecting chickens in the immunovariability it displays among isolates from different geographical areas. *Eimeria maxima* oocysts (named EmaxAPU3) were isolated late in grow-out (6 weeks) from litter in a commercial broiler operation that was using *Eimeria* vaccination as the coccidiosis control program. Cross-protection studies (n = 4) were conducted in immunologically naïve chickens between EmaxAPU3 and two *E. maxima* lab strains (EmaxAPU1, EmaxAPU2) by immunizing with one *E. maxima* strain and challenging with either the homologous or heterologous *E. maxima*. As measured by oocyst output, immunization with EmaxAPU1 protected against homologous challenge (EmaxAPU1) and against heterologous challenge with EmaxAPU3, but not against EmaxAPU2. Similarly, immunization with EmaxAPU3 protected against homologous challenge (EmaxAPU3) and against heterologous challenge with EmaxAPU1, but not against EmaxAPU2. Immunization of chickens with EmaxAPU2 elicited a protective response against homologous challenge (EmaxAPU2), but not against EmaxAPU1 nor EmaxAPU3. The most plausible explanation for the appearance of this immunovariant late in grow-out is that *E. maxima* APU3 escaped immunity directed to *E. maxima* antigenic types in the commercial vaccine.

## 1. Introduction

Avian coccidia that infect chickens are characterized by intestinal site specificity of sporozoite invasion and a lack of cross-immunity between different *Eimeria* species. Of the seven *Eimeria* species infecting chickens, *E. maxima* is considered extremely important not only because of the site of parasite invasion and development (jejunum) which disrupts nutrient uptake, but also because it predisposes chickens to necrotic enteritis (NE). NE is caused by *Clostridium perfringens* that invade and release toxins (e.g., netB) after *E. maxima* has disrupted the intestinal epithelium. *Eimeria maxima* is particularly disruptive because it has at least four schizogonous cycles and invades the subepithelial layer [1,2]. Phenotypic and genotypic differences have been noted between different *E. maxima* isolates [3,4,5,6]. Of interest is the immunovariability among different geographical isolates which may be due in part to the solid immunity that develops in chickens after a primary *E. maxima* infection [7,8,9]. Immunity to homologous *E. maxima* challenge is achievable with as few as 20 oocysts, with lower doses (5–10 oocysts) requiring an additional 2–3 inoculations to produce solid immunity [8,10,11]. Consistent with this concept of cycling is that immunity to homologous challenge develops more rapidly (1–3 weeks) in chicks allowed to ingest excreted oocysts compared to those exposed to only a single inoculum [8,9]. The strong immunity against subsequent *E. maxima* infection is the basis for live virulent or attenuated (precocious) vaccines [12,13,14,15]. One issue for *Eimeria* vaccine producers is immunovariability among different *E. maxima* (for a review, see [14]). In studies conducted over 35 years ago, authors found that the inoculation of chicks with *E. maxima* Houghton (H) failed to protect these chicks against *E. maxima* Weybridge (W) and vice-versa [10]. The phenomenon of *E. maxima* immunovariability has been corroborated by others studying various *E. maxima* geographic isolates [9,16,17,18,19]. Of interest is that precocious *E. maxima*, those whose patent period is shorter due to the absence of at least one schizogonous stage, lose the ability to cross-protect against the non-attenuated parent [20]. This may be due to a dependency on primary and secondary infection to achieve at least some degree of cross-immunity [4,11]. However, it remains unknown why immunovariant *E. maxima* arise in an *Eimeria* population. The present study may provide a clue as to the reason for the emergence of non-cross reactive *E. maxima*.

## 2. Materials and Methods

### 2.1. Parasites

#### *Eimeria maxima* 

APU1 was isolated over 10 years ago from a commercial broiler farm during an anti-coccidial drug treatment program. *Eimeria maxima* APU2 was isolated 7 years ago from a commercial broiler farm utilizing a coccidiosis vaccine program. Both *E. maxima* strains have been propagated every 3–4 months in susceptible broiler chickens using standard methods as approved by the Beltsville Agricultural Research Center IACUC (Animal Use Protocol no. 22-06). *Eimeria maxima* APU3 was isolated from litter at 43 days of grow-out in a commercial broiler farm that was using a commercial *Eimeria* vaccine for coccidiosis control. Litter samples were sent to our laboratory for analysis because the attending poultry veterinarian observed signs of acute coccidiosis in broiler chickens at a point not normally observed late in grow-out. Examination of fecal material after sucrose flotation on a McMaster chamber [21] revealed a mixture of different *Eimeria* oocysts with high numbers of *E. maxima* oocysts (~2 × 10^3^ oocysts/g). Applying ITS1-PCR [22] to DNA extracted from these purified oocysts showed that samples contained *E. acervulina*, *E. maxima*, and *E. tenella*. The *E. maxima* oocysts were enumerated on a hemacytometer and then isolated by limiting dilution on a 96-well microtiter plate (Nunc) by pipetting 10, 1, or 0.1 oocysts/well. Microtiter wells containing a single large (31 μm × 21 μm) oocyst were collected and used to make a pool of 10 oocysts, which were inoculated into 3 susceptible 1 week-old broiler chickens. Fecal material was collected between 6 and 7 days post-inoculation and processed for *Eimeria* oocysts using standard procedures [23]. Samples containing only *E. maxima* oocysts as judged by microscopy and ITS1-PCR were propagated several more times in susceptible chickens until sufficient pure *E. maxima* APU3 oocysts were produced for cross-immunity studies.

### 2.2. Evaluation of Cross-Protection among E. maxima APU1, APU2, and APU3

Preliminary studies in our laboratory found that 100 *E. maxima* APU1 or *E. maxima* APU2 oocysts was a sufficient immunizing dose to elicit a protective response against homologous challenge. Moreover, this same dose was found by our laboratory and others to be a useful challenge dose for assessing resistance to subsequent infection based on oocyst output. In a series of identical immunization-challenge studies (n = 4), cross-immunity between *E. maxima* APU1, APU2, and APU3 was evaluated using oocyst output as a measure of immunity to homologous or heterologous challenge. HR 708 broiler chicks (n = 3/immunization-challenge combination, Longeneckers Hatchery, Elizabethtown, PA, USA) were immunized with 100 *E. maxima* APU1, APU2, or APU3 oocysts at 0, 7, and 14 days of age and challenged with 100 *E. maxima* APU1, APU2, or APU3 oocysts 7 days after the last immunization (day 21). Immunologically naïve chicks that had been housed in a separate facility and not exposed to *E. maxima* oocysts were also challenged with the same number of *E. maxima* APU1, APU2, or APU3 oocysts. All chicks were housed individually (n = 3/treatment), and total fecal droppings were collected from days 5–8 post-challenge and processed for *E. maxima* enumeration. *Eimeria maxima* counts entailed sucrose flotation and microscopy using a McMaster chamber at 100× magnification. Total oocyst output/chicken was calculated using total volume of the fecal slurry and then mean log values were calculated for statistical comparisons between treatment groups.

### 2.3. Ethics Statement

Animal experiments were performed following the Beltsville Agricultural Research Center (BARC) Institutional Animal Use and Care Committee protocol (22-06). Chickens utilized in this study exhibited no outward signs of severe disease over the course of the study. After the study’s conclusion, all chickens were humanely euthanized; all efforts were made to minimize animal suffering. Research procedures were carried out in accordance with national and institutional regulations.

### 2.4. Statistical Comparisons

The immunization-challenge study using *E. maxima* APU1, APU2, and APU3 was conducted 4 times with mean oocyst output/chick calculated for each treatment group. Average *E. maxima* oocyst output between treatment groups for all 4 studies was compared by one-way ANOVA followed by Tukey’s post-test using InStat Statistics Software version 3.05 (GraphPad Software, Inc., San Diego, CA, USA). Statistical difference was inferred at *p* < 0.05.

## 3. Results

**Figure 1:**
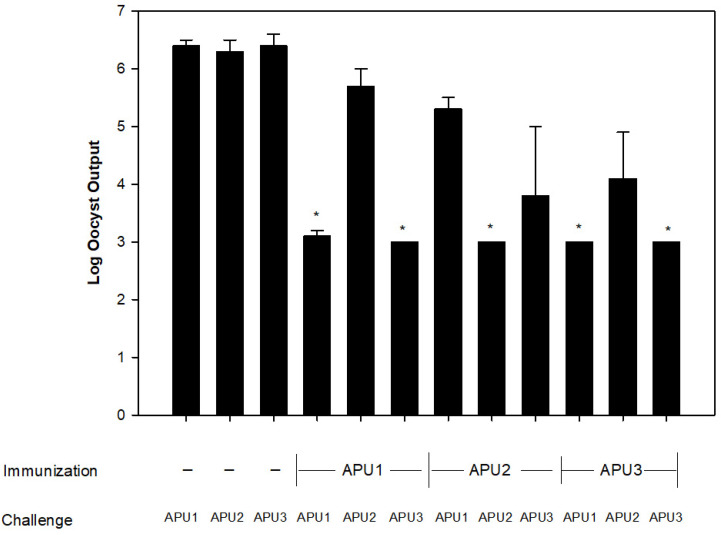
Protection as measured by oocyst output in broiler chickens (n = 3/treatment) immunized with either *Eimeria maxima* APU1, *E. maxima* APU2, or *E. maxima* APU3 oocysts against a homologous or heterologous 100 oocyst challenge infection. Controls were broiler chickens not exposed to *E. maxima* oocysts prior to challenge infection. Average oocyst output was calculated based on means and S.D. of 4 independent studies. Asterisks indicate a significant difference between mean oocyst output of treatment and the respective non-immunized control.

Complete protection against homologous *E. maxima* challenge was observed in all studies. For instance, immunization of chicks with *E. maxima* APU1 led to complete protection against *E. maxima* APU1 challenge (*p* < 0.001) (Figure 1—bar 1 vs. bar 4). Immunization of chicks with *E. maxima* APU2 led to complete protection against *E. maxima* APU2 challenge (*p* < 0.01) (Figure 1—bar 2 vs. bar 8), and immunization of chicks with *E. maxima* APU3 led to complete protection against *E. maxima APU3* challenge (*p* < 0.001) (Figure 1—bar 3 vs. bar 12). Complete cross-protection was also observed between *E. maxima* APU1 and APU3. Primary immunization of chicks with *E. maxima* APU1 completely protected against *E. maxima* APU3 challenge (Figure 1, bar 4 vs. bar 6). The reverse was also seen in chicks immunized with *E. maxima* APU3 were completely protected against *E. maxima* APU1 challenge (Figure 1, bar 12 vs. bar 10). However, immunization with *E. maxima* APU2 conferred no immunity against either *E. maxima* APU1 (Figure 1, bar 8 vs. 7) or *E. maxima* APU3 (Figure 1, bar 8 vs. 9). Likewise, no protection was seen in the reverse order in that immunization with *E. maxima* APU1 (Figure 1, bar 4 vs. 5) or *E. maxima* APU3 (Figure 1, bar 12 vs. 11) did not prevent oocyst development in chicks subsequently challenged with *E. maxima* APU2.

## 4. Discussion

The present study is the first description of an *Eimeria maxima* immunovariant arising during growth of broilers that were vaccinated by spray with a commercial coccidiosis vaccine. This immunovariant, named *E. maxima* APU3, arose 6 weeks after chick placement, which is a time that *Eimeria* oocyst numbers in litter, due to increased immune resistance, are typically low. Oral inoculation of chicks with *E. maxima* APU3 could protect against heterologous *E. maxima* APU1 challenge, but not against *E. maxima* APU2. Although inoculation of chicks with *E. maxima* APU2 was effective against homologous infection, it failed to protect against *E. maxima* APU1 or APU3 challenge. *Eimeria maxima* APU2 is a strain that was isolated early in grow-out from broilers that had been spray-vaccinated with a commercial vaccine. Technology is not available as of yet to discern among these three *E. maxima* strains, and thus it is premature to equate *E. maxima* APU2 with the *E. maxima* present in the commercial vaccine.

Nevertheless, the high numbers of *E. maxima* APU3 (~2 × 10^3^/g) in litter late in grow-out has at least two explanations. One is that *E. maxima* APU3 is distinct from the *E. maxima* present in the coccidiosis vaccine administered at the hatchery. In this scenario, *E. maxima* APU3 existed at low levels in litter at time of chick placement, and its numbers increased over time relative to *E. maxima* in the commercial vaccine. Depending on the coccidiosis control program, *Eimeria* oocyst concentrations typically peak at about 3 weeks after chick placement. It is unusual to observe high concentrations of *Eimeria* oocysts in litter and associated acute coccidiosis in older chickens. The propagation of *E. maxima* APU3 to high numbers may stem from a lack of immunity to it in chicks immunized with another non-cross-reactive *E. maxima* strain. Another possibility, though more remote, is that *E. maxima* APU3 is a de novo genetic variant of the *E. maxima* present in the vaccine. This would entail the vaccine-derived *E. maxima* spontaneously altering its antigenic profile after infecting an immune animal and replicating to high numbers sufficient to cause overt coccidiosis. Although protozoa infecting humans and animals are known to undergo antigen switching, as in *Plasmodium*, *Theileria*, *Babesia*, and *Trypanosoma*, these parasites generally have two-host heteroxenous life cycles [24,25,26,27]. *Eimeria* are monoxenous parasites that produce asexual and sexual developmental stages at fairly specific times after oocyst ingestion. Due to the abundance of immunologically naïve hosts by virtue of the short life-span of commercial broilers and their replacement by 2–3 weeks with another crop of susceptible chicks, *Eimeria* would not be expected to rely on immunovariation for survival. Indeed, our group has found that viable and infectious *Eimeria* oocysts are present in at least 30% of broiler houses at time of chick placement [21]. But it is possible that *E. maxima* also uses immunovariation late in grow-out to ensure survival until the next set of susceptible hosts become available, especially if 70% of poultry houses do not contain measurable numbers of viable *Eimeria* oocysts. Studies are required to determine if those viable *Eimeria* oocysts in litter at placement are antigenic variants of *Eimeria* in the commercial vaccines.

One study found complete cross-immunity among *E. maxima* oocysts recovered from four successive generations of broilers over an 8 mo. period in a single poultry house [28]. However, these oocysts were isolated only once at 4 weeks post-placement from each set of broilers. Thus, the observation of cross-reactive *E. maxima* in this study may be due to the earlier time of oocyst isolation (4 weeks vs. 6 weeks) and that anticoccidial drugs rather than an *Eimeria* vaccine was used for coccidiosis control. Moreover, it is unclear whether chicks were grown on new bedding or used litter, the latter probably containing *E. maxima* oocysts. The authors also concede that the *E. maxima* oocysts used in the immunization-challenge studies were not pure isolates which would complicate any estimate of cross-immunity due to a mixture of cross-reactive and non-cross-reactive *E. maxima*.

In conclusion, our study is the first description of an immunovariant *E. maxima* isolated late in grow-out from a broiler operation utilizing hatchery spray vaccination of chicks with a live *Eimeria* oocyst vaccine. The relationship between the immunovariant and vaccine *E. maxima* is unknown at present, but this study should alert those involved in poultry health to potential issues with vaccination against *E. maxima* to control coccidiosis in broilers.

## Data Availability

The data presented in this study are available on request from the corresponding author.
